# Neuromodulation Treatments Targeting Pathological Synchrony for Tinnitus in Adults: A Systematic Review

**DOI:** 10.3390/brainsci14080748

**Published:** 2024-07-26

**Authors:** Derek J. Hoare, Gillian W. Shorter, Giriraj S. Shekhawat, Amr El Refaie, Bas Labree, Magdalena Sereda

**Affiliations:** 1 NIHR Nottingham Biomedical Research Centre, Hearing Sciences, Mental Health and Clinical Neurosciences, School of Medicine, University of Nottingham, Nottingham NG1 5DU, UK; bas.labree1@nottingham.ac.uk (B.L.); magdalena.sereda@nottingham.ac.uk (M.S.); 2 Department of Speech and Hearing Sciences, University College Cork, T12 EK59 Cork, Ireland; amr.elrefaie@ucc.ie; 3 Drug and Alcohol Research Network, School of Psychology, Queen’s University Belfast, Belfast BT7 1NN, UK; g.shorter@qub.ac.uk; 4 College of Education, Psychology, and Social Work, Flinders University, Adelaide, SA 5001, Australia; giriraj.shekhawat@flinders.edu.au; 5 Tinnitus Research Initiative, Universitätsstrasse 84, 93053 Regensburg, Germany

**Keywords:** acoustic neuromodulation, transcranial direct current stimulation, transcranial alternating current stimulation, vagus nerve stimulation, bimodal stimulation

## Abstract

(1) Background: Tinnitus involves the conscious awareness of a tonal or composite noise for which there is no identifiable corresponding external acoustic source. For many people, tinnitus is a disorder associated with symptoms of emotional distress, cognitive dysfunction, autonomic arousal, behavioural changes, and functional disability. Many symptoms can be addressed effectively using education or cognitive behavioural therapy. However, there is no treatment that effectively reduces or alters tinnitus-related neurophysiological activity and thus the tinnitus percept. In this systematic review, we evaluated the effectiveness of neuromodulation therapies for tinnitus that explicitly target pathological synchronous neural activity. (2) Methods: Multiple databases were searched for randomised controlled trials of neuromodulation interventions for tinnitus in adults, with 24 trials included. The risk of bias was assessed, and where appropriate, meta-analyses were performed. (3) Results: Few trials used acoustic, vagal nerve, or transcranial alternating current stimulation, or bimodal stimulation techniques, with limited evidence of neuromodulation or clinical effectiveness. Multiple trials of transcranial direct current stimulation (tDCS) were identified, and a synthesis demonstrated a significant improvement in tinnitus symptom severity in favour of tDCS versus control, although heterogeneity was high. (4) Discussion: Neuromodulation for tinnitus is an emerging but promising field. Electrical stimulation techniques are particularly interesting, given recent advances in current flow modelling that can be applied to future studies.

## 1. Introduction

Tinnitus is defined as “the conscious awareness of a tonal or composite noise for which there is no identifiable corresponding external acoustic source” [1]. It is often described as a hissing, ringing, whooshing, or buzzing sound and is thought to result from abnormal neural activity in the auditory pathway erroneously interpreted as sound. Subjective tinnitus, where the sound is only heard by the person experiencing it and no source of the sound is identified, affects 14% of the general population, with higher prevalence rates in older adults [2].

Many people with tinnitus report it to be troublesome. To distinguish tinnitus that is experienced from that which is troublesome, the term tinnitus disorder was recently introduced, defined as tinnitus “when associated with emotional distress, cognitive dysfunction, and/or autonomic arousal, leading to behavioural changes and functional disability” [1]. In about 90% of cases, tinnitus is co-morbid with hearing loss, which may confound the disabling effects of tinnitus [3]. An important implication in clinical research is that outcome measures need to distinguish benefits specific to improved hearing from those specific to tinnitus.

Currently, no single treatment has a replicable long-term effect that reduces tinnitus perception. The clinical management of tinnitus is largely based on the impact of symptoms, for example, the use of cognitive behavioural therapies to reduce its intrusiveness or the negative automatic thoughts and emotional reactions that are seen in tinnitus disorder [4,5,6]. Other approaches include neuromodulation therapies that aim to interrupt the pathological neuronal activity associated with tinnitus in a sustained way. In theory, this should lead to a reduction in tinnitus-related emotional distress, cognitive dysfunction, autonomic arousal, and the associated behavioural changes and functional disability.

It is not clear what neurophysiological change is essential for a sustained change in tinnitus perception. Using magnetoencephalography (MEG), Weisz et al. [7,8] found a decreased power in the alpha band (8 Hz to 12 Hz) and increased oscillatory power in both the delta (1–3 Hz) and gamma frequency bands (40–90 Hz), in people with tinnitus, compared to no-tinnitus controls. The differences between the groups were most pronounced in the temporal regions. In a following study, they found a reduced delta but not gamma band activity during residual inhibition [9]. Adjamian et al. [10], also using MEG, reported a higher delta band power in adults with tinnitus and normal hearing than control participants who had no tinnitus or hearing loss. They also found that masking tinnitus reduced delta band activity, and that gamma band activity did *not* correlate with tinnitus or hearing loss. Schlee et al. [11] examined auditory alpha activity using MEG, finding it was reduced in participants who had tinnitus. They further demonstrated an association between differences in alpha band activity and the duration of tinnitus. Electroencephalography (EEG) studies corroborate some but not all these effects. Tass and Popovych [12] associated residual inhibition (a reduced tinnitus percept achieved using external acoustic stimulation) with reduced delta band power, whereas Ashton et al. [13] found gamma power activity was higher in participants with tinnitus but delta and alpha band power was unaltered. Taken together, changes in delta band oscillatory activity appear to provide the best objective predictor of changes in tinnitus. That said, different neuromodulation interventions act via different neural mechanisms. For example, transcranial direct current stimulation (tDCS) is hypothesised to have a sustained polarising or depolarising effect on neuronal membrane potentials and to modulate gamma band activity, whereas transcranial alternating current stimulation (tACS) is thought to modulate neural activity by up- and down-regulating synapses and cause changes in alpha band activity [14,15,16].

Acoustic neuromodulation therapies to affect changes in cortical oscillations emerged from work on Parkinson’s disease using ‘co-ordinated reset’ (CR^®^, Cologne, Germany) technology [17]. CR^®^ uses repeated patterns of stimulation to change the mean synaptic weight and is hypothesised to reset oscillatory dynamics over time, returning the brain to its normal desynchronised (at rest) activity state [12]. In an animal model of epilepsy, Tass and Hauptmann [18] reported a sustained desynchronisation between neuronal populations after treatment with multi-site electrical stimulation. They also provided a computational model predicting how patterned sound stimuli could, over time, interrupt the pathological synchronous activity associated with tinnitus in a sustained way [12].

Various neuromodulation therapies using bimodal (e.g., combinations of acoustic/electrical/somatosensory stimuli) techniques have also been developed from proof-of-concept studies in animals. Engineer et al. [19] demonstrated how vagus nerve stimulation (VNS) paired with acoustic stimuli (multiple tones outside the presumed tinnitus frequency) either reversed or prevented both the physiological and behavioural evidence of tinnitus in an animal model of noise-induced tinnitus. In that treatment, acoustic stimuli were considered necessary to target the auditory cortex, and VNS necessary to promote plastic change through the “synergistic action of multiple neuromodulators” [19]. Acetylcholine was also hypothesised to play an important role [20]. Translating this to humans, De Ridder et al. [21] implanted electrodes on the left vagus nerve of 10 patients with severe tinnitus disorder. VNS paired with tones was delivered for 2.5 h daily for 20 days. Some patients reported reduced tinnitus severity immediately post-treatment, and (EEG) delta and theta band power was decreased in the treatment condition compared to sham. A non-invasive version using transcutaneous VNS (tVNS) and notched music also showed proof of concept [22,23].

The objectives of this review were to (1) evaluate the effectiveness of neuromodulation therapies that explicitly target pathological synchronous activity to reduce tinnitus and thus tinnitus disorder, (2) evaluate their effectiveness in terms of reduced co-morbid symptoms and improved quality of life, and (3) review the physiological changes and adverse effects associated with different neuromodulation (desynchronisation) therapies.

## 2. Materials and Methods

This review was originally initiated as a Cochrane Ear, Nose, and Throat Disorders Group (CENTDG) review [24] and registered on PROSPERO (CRD42015027131). However, it was paused to conduct other reviews as part of a suite [25], and CENTDG was subsequently disbanded, so the review was produced independently but retained most of the Cochrane methodology.

### 2.1. Inclusion/Exclusion Criteria

We included randomised controlled trials involving adults (≥18 years) with subjective tinnitus. Interventions were therapies that were explicitly hypothesised to modulate pathological synchronous neural activity associated with tinnitus, to deliver a sustained desynchronisation of that activity. That included several forms of neuromodulation intervention, including tDCS, tACS, VNS, tVNS, cochlear implantation, acoustic neuromodulation therapy, and paired electrical/somatosensory and acoustic stimulation therapies. Trials of tinnitus interventions that were categorised as neuromodulation but did not specify targeting pathological synchronous activity were excluded. Studies involving children or objective tinnitus were excluded. There were no restrictions on language or date.

### 2.2. Search Strategy

Ovid (Medline, EMBASE, CAB Abstracts, Amed), PubMed, Web of Knowledge, Web of Science, and the Cochrane study databases were searched using the search terms tinnitus AND (tdcs OR neuromodulation OR coordinated reset OR desynchronisation OR ((transcranial OR transcutaneous) AND stimulation) OR electrostimulation OR neurostimulation). For an example search, see Box A1. Searches were initially conducted by the CENTDG Information Specialist up to January 2023 and were updated by DJH, with the final searches conducted in March 2024.

We scanned the reference lists of identified publications for additional trials and contacted the trial authors where necessary. In addition, Ovid Medline was searched to retrieve existing systematic reviews published up to February 2023 relevant to this systematic review, so that we could scan their reference lists for additional trials.

### 2.3. Selection of Studies

Pairs of authors independently reviewed each study to determine its eligibility for inclusion. Title and abstracts were screened first, and any records that potentially met inclusion criteria were taken forward to a full-text screening. Any disagreements between authors were discussed until a consensus was reached.

### 2.4. Data Extraction

Data were extracted using a purposefully designed data extraction form that was piloted on a subset of the included records. The information extracted included the following: trial design, setting, methods or randomisation and blinding, power, inclusion and exclusion criteria, type of intervention and control, treatment duration, baseline characteristics of participants (age, sex, duration of tinnitus, tinnitus severity, tinnitus loudness and pitch estimates, details of co-morbid hearing loss, anxiety or depression), treatment fidelity, type and duration of follow-up, outcome measures and statistical tests, and details of any attrition or exclusion. The outcome data extracted were the group mean and standard deviation at pre- and post-intervention and follow-up, and the results of any statistical tests of between-group comparisons.

Where not reported or otherwise available, we estimated standard deviations using the available data, such as standard errors, confidence intervals, *p* values, and t values. Where the data were only available in graphs, the authors made and agreed numeric estimates. After independent data extraction by pairs of authors, the extracted data were reviewed for disagreements and revisited and discussed as required, to reach a consensus on the dataset.

### 2.5. Risk of Bias

The risk of bias of the included trials was assessed using the Cochrane risk of bias tool [26]. The risk of bias was assessed as a ‘low’, ‘high’, or ‘unclear’ risk of bias for sequence generation; allocation concealment; blinding; incomplete outcome data; selective outcome reporting; and other sources of bias. Pairs of authors independently assessed the risk of bias on each record and met to discuss any disagreements and reach an agreed single judgement for each factor and each study. In some instances, additional authors were consulted to ensure the consistency of judgements across all authors.

### 2.6. Analysis

The primary comparison in this review was neuromodulation therapy versus a placebo or no-intervention control. Of secondary interest was the relative effectiveness of individual neuromodulation therapies compared to each other or to standard therapy options, including hearing aids, sound generators, psychological or education-based therapies, or combinations of therapies.

The primary outcomes were (1) tinnitus symptom severity (e.g., impact on quality of life, activities of daily living, sleep), measured as the global score on a multi-item tinnitus questionnaire, and (2) serious adverse effects. The secondary outcomes were (3) generalised anxiety, (4) generalised depression, (5) generalised quality of life, all measured using multi-item questionnaires, (6) neurophysiological change (as measured by MEG or EEG), and (7) adverse effects.

Continuous outcomes were summarised as the mean difference (MD) with 95% CI. The standardised mean difference (SMD) (Cohen’s d effect size (ES)) was used when different questionnaire measures were used for the same outcome. The heterogeneity of aggregated effect sizes was calculated using Cochran’s Q statistic (Chi^2^ test with K-1 degrees of freedom, where K is the number of studies) and the *I*^2^ statistic (percentages of around 25%, 50%, and 75% of *I*^2^) would mean a low, medium, and high heterogeneity, respectively [27].

Different forms of neuromodulation therapy were analysed separately. Where more than one study was identified for a given form of neuromodulation therapy, and the synthesis of the data was appropriate, RevMan 5.3 was used to perform meta-analyses. Data from randomised controlled trials were pooled using a fixed-effect model, except when heterogeneity was found. A sensitivity analysis was performed to explore whether heterogeneity was a result of a high risk of bias. Due to the number of studies included in meta-analyses, this was only applied to the synthesis of data for the effect of tDCS on tinnitus symptom severity.

## 3. Results

**Figure 1:**
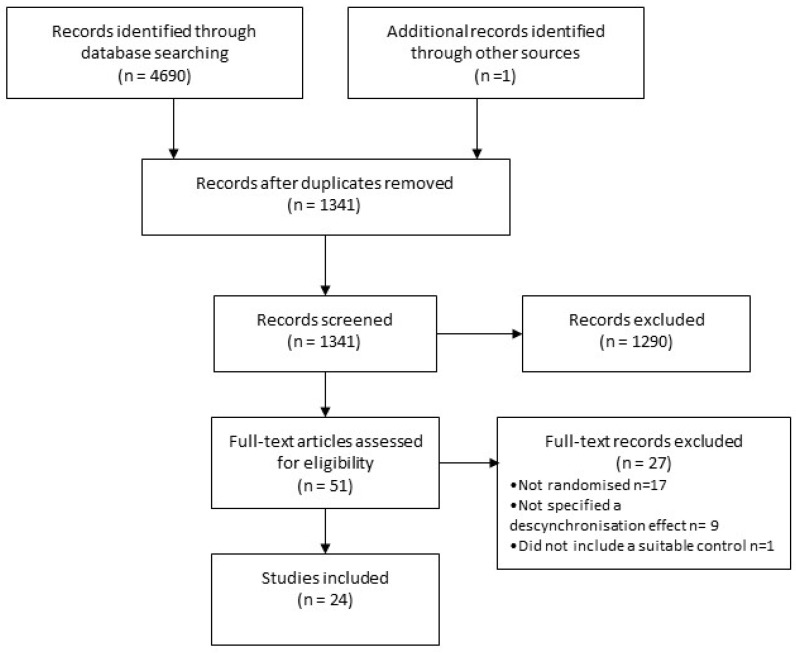
PRISMA chart showing flow from record identification to final inclusion in review of neuromodulation treatments for tinnitus.

A flowchart of study retrieval and selection is provided in Figure 1. Our electronic database searches identified 4690 records, and one additional record was identified through a Google Scholar alert. Of those, 1341 remained after removing duplicates, all of which were screened. Twenty-four RCTs met our criteria for inclusion [28,29,30,31,32,33,34,35,36,37,38,39,40,41,42,43,44,45,46,47,48,49,50,51].

For a summary description of each study, including details of dosage and duration, see Table 1. All studies recruited adult participants (18 years or over). The mean age of the participants included in the studies ranged from 41.1 years to 59.9 years. Of those reported, 39.7% of participants were female and 60.3% were male. Males accounted for between 33 and 100% of participants, depending on the study. Only one small study had an equal number of male and female participants [30].

Most studies recruited participants with chronic tinnitus; however, the definition of chronic tinnitus differed from, for example, ‘tinnitus as lasting 3 months or over’ [43,44,46], to ‘6 months or over’ [29,33,35,36,37,45,47,50], ‘1 year or over’ [28,40,48,49], and ‘more than 2 years’ [32,41,51]. One study did not specify the minimum duration for chronic tinnitus [30]. Garin et al. [34] recruited participants with tinnitus stable for at least 2 months and Mei et al. [39] those with tinnitus recurrent for 1 month or stable for 5 days. Dobie et al. [32] did not specify any inclusion criterion related to the duration of tinnitus.

Several studies recruited participants specifically with tonal tinnitus [35,44,45,46,47,49]. Four studies had specific inclusion criteria regarding the dominant tinnitus frequency which were a dominant tinnitus frequency between 1 and 12 kHz [44], below 9 kHz [46], and between 0.2 and 10 kHz [35,47]. Some studies had inclusion criteria regarding hearing loss, e.g., sensorineural hearing loss [30]; some with a specific pure tone average (PTA) requirement, e.g., below 60 dB HL in the ear with tinnitus [35], or below 70 dB HL for frequencies of one-half octave above and below the tinnitus frequency [44]; or a maximum hearing impairment of 50 dB [45]. Forogh et al. [33] specified an exclusion criterion of fluctuating hearing loss.

A few studies also had an inclusion criterion requiring a minimum level of tinnitus symptom severity, e.g., tinnitus severe enough to justify the patients’ expenditure of time [32], or a minimum score on a multi-item tinnitus questionnaire [35,41]. Most studies reported tinnitus severity scores at baseline and post-intervention/follow-up using established multi-item tinnitus questionnaires, the Tinnitus Functional Index (TFI, [52], the Tinnitus Handicap Inventory (THI, [53]), the Tinnitus Handicap Questionnaire (THQ, [54]), or the Tinnitus Questionnaire (TQ, [55]).

Some studies had eligibility criteria based on mental and emotional state, e.g., excluding participants with current or a history of mental disorders such as severe depression or anxiety [30,35,39,40,44,45,46,50] or significant scores on indicative questionnaires such as the Beck Depression Inventory (BDI [56]) [49]. Others did not have such exclusions, and some reported baseline anxiety or depression scores. Dobie et al. [32] found that 17 out of 20 participants reported depression or anxiety, which they blamed on their tinnitus. Baseline scores in several studies indicate that many participants might have suffered from some level of either depression or anxiety [40,46,48,50].

### 3.1. Risk of Bias in the Included Studies

**Figure 2:**
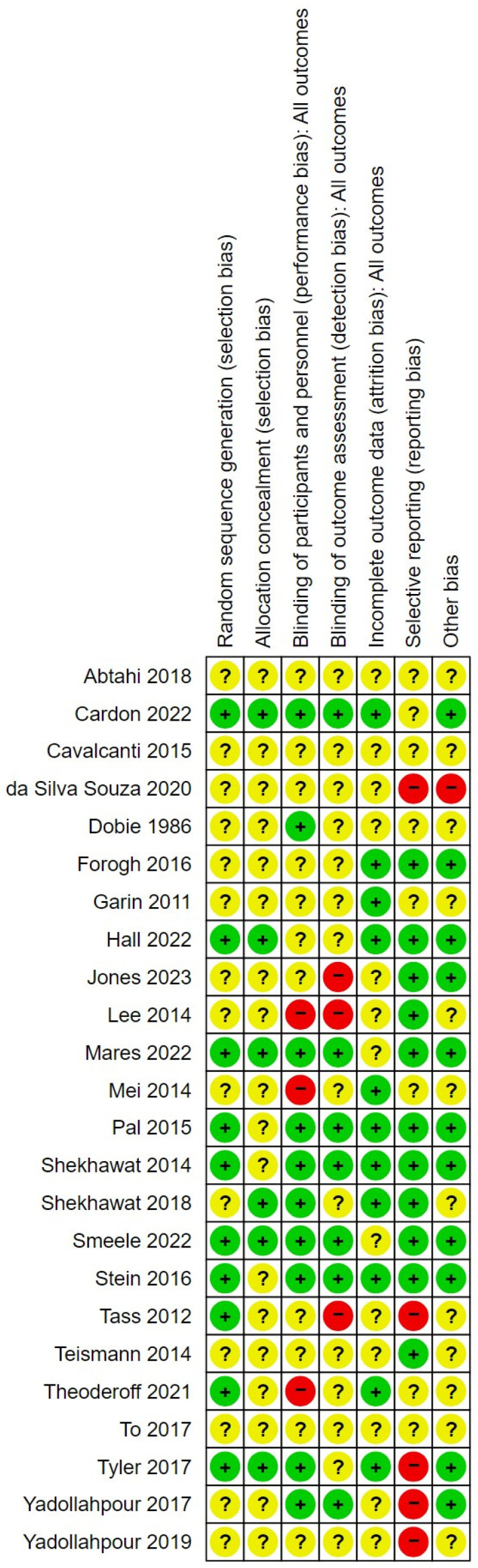
Risk of bias summary for review of neuromodulation treatments for tinnitus [28,29,30,31,32,33,34,35,36,37,38,39,40,41,42,43,44,45,46,47,48,49,50,51].

The risk of bias in the studies is summarised in Figure 2. For most sources of bias in most studies, the risk could not be confirmed as low or high due to a lack of reporting in the clinical trial registration, protocol or published study record. All studies were included on the basis they were randomised controlled trials; however, only 10 studies provided details of the methods used to undertake randomisation, and only six studies provided details of the steps undertaken to ensure allocation concealment. The studies that did report these details tended to report well, were more recent, and overall had a lower risk of bias than the other studies [29,38,43]. That said, the most recent included study [36] had an unclear or high risk of bias across most sources.

Nine studies were judged to have a high risk of bias from one or more sources. For example, Jones et al. [36] was judged to have a high risk of bias for detection bias, because the trial registration described a quadruple blinding including outcome assessors, whereas the study publication reports only double blinding. In Lee et al. [37], there was a high risk of performance and detection bias, as neither the study personnel nor outcome assessors were blinded.

There was a high risk of reporting bias in five studies, for various reasons. For example, DaSilva Souza et al. [31] did not report a planned secondary outcome (acuphony) listed in the trial registration but did report EEG data which was not listed as an outcome in the trial registration. In the case of Tass et al. [45] a trial registration was available, but this did not define any planned outcomes or analyses, and the trial entry criteria did not match the registration information.

### 3.2. Synthesis of Findings

#### 3.2.1. Acoustic Neuromodulation Interventions

**Figure 3:**
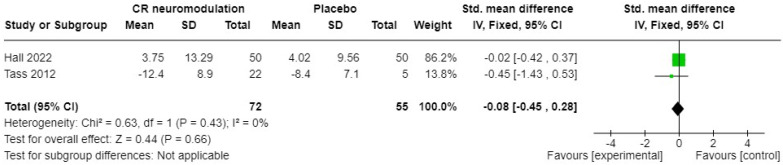
Forest plot of acoustic CR neuromodulation versus placebo effects on tinnitus symptom severity [35,45]. Green box indicates relative size of sample and mean effect. Diamond indicates pooled effect.

Two studies of acoustic CR(r) neuromodulation [35,45] versus a placebo sound measured tinnitus symptom severity pre- and post-intervention, using the THQ or TQ. The standardised mean difference (SMD) and 95% confidence intervals (CIs) are shown in Figure 3. A fixed-effects meta-analysis showed no significant overall difference between acoustic CR(r) neuromodulation and the placebo sound (SMD −0.08, 95% CI −0.45 to 0.28); 127 participants in total. The heterogeneity (I2) was zero, so not significant (Chi2 = 0.63, df = 1, *p* = 0.43). A further study under the name Desyncra™ [47] compared this acoustic treatment to cognitive behavioural therapy (CBT) for tinnitus in a randomised trial stratified by hearing aid use. A sample size of 200 was originally estimated, but only 61 participants took part. The primary outcome was change in TQ score after 24 weeks, and there was no difference between groups, suggesting the superiority of CBT as the less intensive treatment (6 h of CBT versus 24 weeks of device use for 4–6 h per day).

One study of tailored notched music training [44] measured tinnitus symptom severity pre- and post-intervention, using the THQ as the primary outcome measure. A mixed-model ANOVA revealed no significant difference between tailored notched music training and a music training using a ‘roving’ notch (F (1,80) = 0.678, *p* = 0.794). The TQ was also used, as a secondary measure, and this comparison was also not significant (F(1, 80) = 0.973, *p* = 0.837). Also in this category, one study of high-definition infraslow pink noise stimulation [43] measured tinnitus symptom severity as a secondary measure pre- and post-intervention using the TQ and the TFI. The data were used for descriptive purposes only, as the study was considered a pilot, with primary outcomes of safety and feasibility. No serious adverse effects were reported in studies of acoustic neuromodulation interventions.

Regarding secondary measures of interest, only Smeele et al. [43] measured generalised anxiety and generalised depression pre- and post-intervention, using the DASS, but these data were for descriptive purposes only. One study [35] measured generalised quality of life, pre- and post-intervention, using the World Health Organization Quality of Life tool [57]. The difference between the intervention and control was not significant (MD = −0.1, 95% CI −0.36 to 0.16; 48 participants). Smeele et al. [43], used both the WHO-5 [58] and the EQ-5D-5L [59] but for descriptive purposes only.

**Figure 4:**
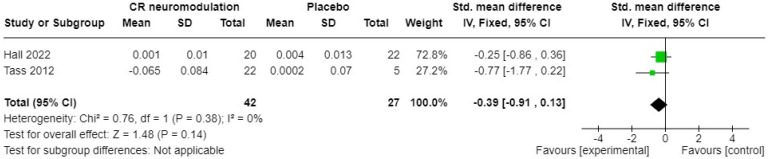
Change in oscillatory power in delta brainwave patterns as measured by electroencephalography in acoustic CR neuromodulation versus placebo control [35,45]. Green box indicates relative size of sample and mean effect. Diamond indicates pooled effect.

Both placebo-controlled studies of acoustic CR(r) neuromodulation [35,45] measured neurophysiological change after intervention using EEG. Both studies reported a change in normalised oscillatory power in the delta brainwave pattern. The standardised mean differences (SMDs) and their 95% confidence intervals (CIs) are shown in Figure 4. A fixed-effects meta-analysis showed no significant overall difference between acoustic CR(r) neuromodulation and placebo sound (SMD −0.39, 95% CI −0.91 to 0.13). The heterogeneity (*I*^2^) was zero, so not significant (Chi^2^ = 0.76, df = 1, *p* = 0.38).

Smeele et al. [43] conducted resting-state electroencephalography (rsEEG) pre- and post-intervention and found a decreased beta-1 activity in the posterior cingulate cortex post-intervention in the control condition, whereas the active treatment group showed decreased beta-1 activity in the inferior parietal lobule post-intervention. An increased functional connectivity in the beta-1 frequency between the left and right parahippocampus (PHC) was also found in the active treatment group compared with controls post-intervention (*p* = 0.01; t = 4.24), although this was not observed at a later follow-up.

Some adverse effects of acoustic CR(r) neuromodulation were reported by Hall et al. [35]. Of their 100 participants, two were withdrawn from the treatment arm of the RCT within 2 weeks of being fitted, because they reported their tinnitus had worsened and had become unbearable. Two further participants were withdrawn during the subsequent open phase of the trial for the same reason. Theodoroff et al. [47] also reported the adverse effects of Deyncra™. Of the 26 participants in their sound therapy arm, one participant experienced exacerbated hyperacusis symptoms and 10 reported their tinnitus becoming louder after using the device. All adverse effects reportedly resolved with a treatment break within 3 weeks, or in the case of hyperacusis, by the end of the trial.

Adverse effects (reported as harm) in the single study of tailored notched music training were recorded by Stein et al. [44] and included consistently or occasionally louder tinnitus (*n* = 7), the addition of new tinnitus sounds (*n* = 2), increased tinnitus awareness (*n* = 2), and psychological stress (*n* = 1) in the treatment group. Adverse effects were more frequently reported by participants in the control group than in the treatment group. Smeele et al. [43] reported various adverse effects in both their treatment and control conditions. Dizziness (*n* = 3), sore eyes (*n* = 2), dry mouth, itchy scalp, and earache (*n* = 1) were more often or only reported in the treatment group.

In summary, acoustic neuromodulation interventions have only been investigated in a few studies. They appear to be safe, with no serious adverse effects reported. A transient increase in tinnitus loudness appears to be common, however. Some neurophysiological effects of acoustic treatments have been reported; these do not correspond to changes in self-reported tinnitus symptom severity, and, when measured at follow-up, are not themselves sustained.

#### 3.2.2. Transcranial Direct Current Stimulation

**Figure 5:**
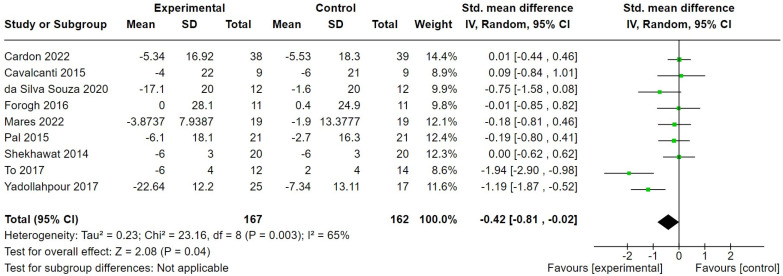
Forest plot comparing effects of multiple sessions of transcranial direct current stimulation versus sham stimulation on tinnitus symptom severity [29,30,31,33,38,40,41,48,50]. Green box indicates relative size of sample and mean effect. Diamond indicates pooled effect.

Nine studies of tDCS [29,30,31,33,38,40,41,48,50] used multiple sessions and measured tinnitus symptom severity pre- and post-intervention, using a multi-item tinnitus questionnaire (TFI, THI, THQ, or TQ), so these data were pooled. We pooled studies regardless of whether they used conventional or ‘high-definition’ tDCS, given the lack of evidence of a difference in effect between the two methods [60]. The SMDs and their 95% CIs are shown in Figure 5. A random-effects meta-analysis showed a significant overall difference between tDCS and the control (eightsham tDCS, one waitlist control) (SMD −0.42, 95% CI = −0.81 to −0.02); 329 participants in favour of tDCS. The heterogeneity was high, however (*I*^2^ = 0.65, Chi^2^ = 23.16, df = 8, *p* = 0.003). A sensitivity analysis removing only one study judged at the highest risk of bias [31] rendered the comparison not significant (SMD −0.38, 95% CI = −0.81 to 0.04); 305 participants, whilst the heterogeneity was unaffected.

One further study [51] of tDCS measured tinnitus symptom severity pre-and post- single sessions of TDCS in a cross-over randomised trial, but only pooled data were available. Another study from Dobie et al. [32] was multisession, but the data were unavailable. One study [46] compared tDCS combined with tailored notched music to a sham tDCS combined with tailored notched music but found no effect of condition or interaction effect in the THQ, THI, or TQ scores (ANOVA, *p* > 0.05 in all cases).

Three studies evaluated tinnitus symptom severity at follow-up. Forogh et al. [33] evaluated tinnitus symptom severity at 2 weeks follow-up, finding no significant effect. Pal et al. [40] conducted follow-up assessments at 5 days, 1 month, and 3 months after intervention, and again found no effect of condition or interaction effect (repeated measures ANOVA, *p* = 0.3228 and *p* = 0.6926, respectively). Mares et al. [38] conducted follow-up assessments 6 weeks and 6 months after intervention but found no significant differences in tinnitus symptom severity between groups.

Serious adverse effects associated or potentially associated with tDCS or sham tDCS were reported by Shekhawat and Vanneste [18]. Scalp burns were reported by eight participants, one of which (experienced by a participant in the sham condition) was rated as severe. No other study of tDCS reported serious adverse effects.

**Figure 6:**
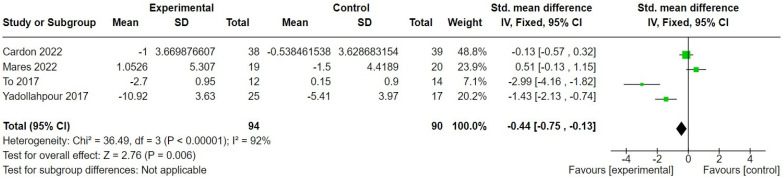
Forest plot comparing change in generalised anxiety post-transcranial direct current stimulation versus sham control [29,38,48,50]. Green box indicates relative size of sample and mean effect. Diamond indicates pooled effect.

In terms of secondary outcomes, four studies [29,38,48,50] measured generalised anxiety, pre- and post-intervention, using either the Hospital Anxiety and Depression Scale (HADS, [61]) or Beck Anxiety Inventory (BAI, [62]). The difference between intervention and control was significant in favour of the intervention (SMD = −0.44, 95% CI −0.75 to −0.13); 184 participants (Figure 6, although the heterogeneity was extremely high (92%).

**Figure 7:**
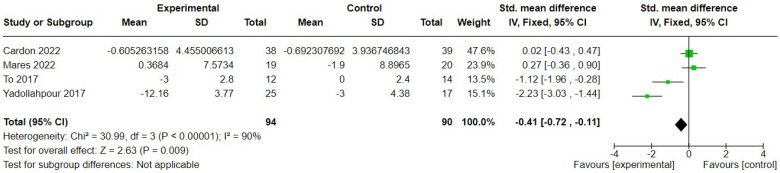
Forest plot comparing change in generalised depression post-transcranial direct current stimulation versus sham control [29,38,48,50]. Green box indicates relative size of sample and mean effect. Diamond indicates pooled effect.

The same four studies [29,38,48,50] also measured generalised depression, pre- and post-intervention, using the BDI, the Zung Self-Rating Depression Scale (SDS, [63]), or the HADS. The difference between treatment and control was significant in favour of treatment (SMD = −0.4, 95% CI −0.72 to −0.11), 184 participants (Figure 7). To et al. [48] additionally measured the change in generalised depression using the HADS as a second measure of depression but found no significant difference between groups (MD = −0.74, 95% = −1.18 to 0.3), and when their HADS data were substituted for BDI data in a meta-analysis, the SMD was not significant (SMD = −0.57, 95% CI = −1.55 to 0.42), 184 participants.

The generalised quality of life was measured in one study of tDCS. Mares et al. [38] reported WHO–Quality of Life–Bref [64] domain scores pre- and post-intervention and at follow-up. The only significant result was immediately post-intervention on Domain 2, which measures psychological wellbeing (U = 96, *p* = 0.006; adjusted *p* = 0.023), compared to the sham. However, none were significant at follow-up.

Neurophysiological change was measured in one study of tDCS [31]. An EEG source localisation analysis after intervention showed that the active tDCS group presented significantly lower theta (t = 0.876; *p* < 0.05) and beta-1 (t = 0.821; *p* < 0.05) bands than the placebo in an eyes-open condition. In an eyes-closed condition, however, there were no statistically significant differences between tDCS and controls after intervention in any of the frequency bands.

Adverse effects with tDCS or sham tDCS were reported by Shekhawat and Vanneste [42]. Participants in the tDCS group reported headache (*n* = 2), neck pain (*n* = 2), scalp pain (*n* = 3), sleepiness (*n* = 6), and trouble concentrating. Participants in the sham condition reported scalp pain (*n* = 3), sleepiness (*n* = 4), and acute mood changes (*n* = 1). These were experienced during stimulation, but none were significant enough to stop the stimulation. Abtahi et al. [28] also reported adverse effects. Of the participants receiving tDCS, one reported headache and one reported insomnia. Participants receiving sham tDCS reported no adverse effects. Garin et al. [34] reported that one participant was excluded after their first treatment session as she required drug treatment for depression. Otherwise, the adverse effects associated with tDCS included transient tinnitus worsening (four participants) or slight pain (one participant). Mares et al. [38] reported adverse effects in 5.3% of active and 5.8% of sham control sessions. These included itching, a burning sensation, fatigue, tingling, headache, and exacerbation of tinnitus. Cardon et al. [29] reported that eight participants (10.4%) experienced side effects. In the active HD-tDCS group, two participants reported light, transient headaches after the stimulation sessions. In the sham control group, four participants reported light, transient headaches, one reported more serious migraine-like headaches, and one reported tingling sensations in their extremities after each session. No significant differences in the presence of side effects were found between the active and control groups (*p* = 0.18). In contrast, To et al. [48] reported there were no adverse effects of the treatment.

Two studies only reported adverse effects on tinnitus; Forogh et al. [33] report tinnitus worsening in both the active and sham treatment groups. In the treatment group, four out of 11 participants experienced worsening tinnitus. One participant discontinued the intervention after one session. Three participants experienced worsening tinnitus after completing 5 days of treatment. For one of those participants, the severity of tinnitus returned to baseline within 5 weeks. For the other two, however, the effect persisted for at least 19 weeks after treatment. Teismann et al. [46] reported one patient (of 21) withdrew from the study due to new tinnitus sounds arising in treatment.

In summary, multiple controlled trials of tDCS for tinnitus have been conducted and overall appear to have a small but significant acute effect on tinnitus symptom severity. At the same time, multiple transient adverse effects of tDCS or sham tDCS were reported, most consistently an increase in tinnitus symptom severity or the emergence of additional tinnitus sounds. The heterogeneity was high across these studies, and in the few studies that measured tinnitus at follow-up, the effect on tinnitus symptom severity does not appear to be sustained. Generalised anxiety and depression also appear to improve acutely, although the number of studies that measured these variables is small and the heterogeneity high.

#### 3.2.3. Vagus Nerve Stimulation

A single study of vagus nerve stimulation paired with sound [49] measured tinnitus symptom severity pre- and post-intervention using the THI, THQ, and TFI. Although a primary outcome measure was not specified (the trial was described as a pilot), the analysis of THI data was fully reported. A repeated measures ANOVA of paired vagus nerve stimulation and the unpaired use of vagal nerve stimulation and sound revealed no significant effect of condition (F = 0.001, *p* = 0.97) or time/condition interaction effect (F = 0.10, *p* = 0.91). The summary analyses provided for the THQ and TFI were also not significant.

A single study of vagus nerve stimulation with auricular acupuncture and sound masking [39] measured changes in tinnitus severity category (as calculated from THIS scores), pre- and post-intervention. After 4 weeks of treatment, there was no difference in the numbers of participants in different severity categories between those receiving vagus nerve stimulation with auricular acupoint and those in the control group (flunarizine hydrochloride and oryzinol) (*X*^2^ = 1.981, *p* = 0.16). After 8 weeks of treatment, however, there were significantly more participants in the milder category of tinnitus severity in the intervention group (*X*^2^ = 9.315, *p* = 0.002). No significant adverse effects were reported in either study of vagus nerve stimulation.

In terms of secondary outcomes, generalised anxiety and generalised depression were measured in Tyler et al. [49] but were not reported. Generalised quality of life and neurophysiological changes were not measured in either study of vagus nerve stimulation. Adverse effects in the single study of paired vagus nerve stimulation [49] were described as mild, moderate, and well tolerated. Two participants experienced iatrogenic vocal cord paralyses (hoarseness or voice weakness) lasting for more than 12 weeks after implantation. In one of those participants, vocal cord paralysis did not recover completely, although they declined treatment or therapy for it. The implant failed in one participant, requiring replacement surgery. Mei et al. [39] did not report any adverse effects.

#### 3.2.4. Alternating Current Stimulation

A single study of alternating current stimulation [37] measured tinnitus symptom severity pre- and post-intervention using the THI. The change in tinnitus symptom severity was significantly greater in the intervention group than the control group (MD = −7.3, 95% CI −11.25 to −3.36; 65 participants). No significant adverse effects were reported. Generalised anxiety, generalised depression, generalised quality of life, and neurophysiological changes were not measured by Lee et al. [37], and no adverse effects were reported.

#### 3.2.5. Combined Auditory and Somatosensory Stimulation

One study involved an intervention combining auditory and somatosensory stimulation. Jones et al. [36] compared precisely timed bi-sensory auditory and somatosensory stimulation to auditory stimulation only. Tinnitus symptom severity was measured using both the TFI and THI. They found a statistically significant decrease on both measures post-intervention and at a 6-week follow-up (*p* = 0.018 to <0.001) in favour of the combined stimulation protocol. No other outcomes of interest were measured in this study.

## 4. Discussion

Recent decades have seen the translation of neuromodulation techniques into clinical alternatives to pharmacological treatment of neurological and neuropsychiatric disorders [65]. This systematic review assessed the effects of neuromodulation treatments for tinnitus. We specifically included the subset of treatments that are hypothesised to affect changes in cortical oscillatory activity, and thereby reverse or alter the pathological synchronous activity associated with tinnitus. The outcomes of interest in this review were tinnitus symptom severity, depression, anxiety, quality of life, oscillatory power, and adverse effects. Twenty-four studies across five distinct forms of treatment were included.

Evidence for the effectiveness of acoustic neuromodulations therapies was very limited, with most treatments showing equivalence to their well-matched controls, despite some evidence that they affected neurophysiological changes. Where the control condition used sound stimuli as a ‘placebo’, it may be that they are exacting some other form of neurophysiological change and/or clinical benefit than the treatment condition. Waitlist or no-intervention control groups appear warranted in that case. A similar scenario is seen in sound-based treatments for tinnitus that are standard care, such as hearing aids, combination hearing aids, and ear level sound generators [66,67]. None have shown superiority in the limited number of clinical trials that have been conducted, and none of those trials included a waitlist control group, placebo, or education/information only. Given the evidence, the choice to prescribe or use acoustic therapies should come down to professional experience and patient preference.

Other forms of neuromodulation techniques reviewed here included tACS, VNS, and bimodal acoustic and somatosensory treatments. With only single studies, or studies using very different approaches, none were suitable for synthesis. All appeared safe, and tACS and bimodal stimulation showed an improvement of tinnitus symptom severity in single studies. Further studies with improved reporting (discussed later) are warranted.

The largest body of evidence in this review related to tDCS as a treatment for tinnitus. Multiple studies compared active tDCS to a sham control condition involving a brief (30–90 s only) electrical stimulation. The treatment condition across studies varied in the number of sessions, duration of sessions, dose, and electrode montage. The synthesis of the findings was significant for an effect in favour of tDCS, but this was largely driven by two studies with large positive effects sizes, and was not significant when one study with a high risk of bias was removed. Nevertheless, the study of tDCS for tinnitus appears worth pursuing. The three studies showing the highest individual effect sizes (1) used a higher dose stimulus than other studies [50], (2) compared to a waitlist control instead of a placebo/sham [48], and (3) reported significant neurophysiological change (reduced theta and beta-1 band power) post-treatment versus sham [31]. The latter was the only study of tDCS for tinnitus to measure oscillatory power as an outcome, so this warrants replication in future studies. Future studies should also address the issue of dose, duration, number of sessions, electrode montage, etc. Our recent network meta-analysis examined the effects of tDCS stimulation on tinnitus, depression, and anxiety, aiming to identify a set of parameters (dose, duration, number of sessions, electrode montage, etc.) that would be optimal for the treatment of tinnitus [68]. From the evidence available, we predict that between 5 and 10 sessions at 20 min each is likely to be optimal, whereas analysis of the electrode montage data showed that, at least for conventional tDCS, spatial specificity may not be important to treatment effectiveness (and therefore not be a parameter that needs further optimisation). Rather, what needs further research is the optimisation of dose, or more specifically, ensuring a sufficiently strong and correctly distributed electric field to ensure a sufficient stimulation of the intended cortical targets. All previous studies of tDCS for tinnitus have administered the same level of current (1–4 mA) to all participants. We know, however, that anatomical variations (e.g., skull thickness, difference in brain morphology, pronounced anatomical differences between children and adults) influence the amount of current reaching the target brain areas, and thus the effectiveness of tDCS [69]. To overcome this ‘black box’ issue, techniques such as current flow modelling (CFM) can be applied [70]. CFM techniques have advanced to a stage where, based on detailed anatomical scans, it is now possible to estimate the intensity of electrical fields, their spatial extent, and direction of current flow in a target brain region during tDCS. CFM has been shown to have the capacity to eliminate the variance in electrical field intensities at a cortical target site [71]. Whilst the research would be resource intensive, this is an exciting advance and an opportunity for a truly targeted treatment of tinnitus, where the target and the dose of current reaching that target can be meaningfully demonstrated.

There are many issues which will limit confidence in the estimate of the effects reported in this review. By the nature of tinnitus, the samples of individuals are heterogenous in so many ways, from their lived experience to its impact on quality of life and function. Equally, the aetiology of tinnitus will likely differ (e.g., the level and nature of sensorineural hearing loss, presence of otosclerosis or other conditions, whether tinnitus has a somatosensory element to it), and exclusion criteria also differ within and across studies. Differences in the definition, and therefore inclusion criteria of, chronic tinnitus were notable, and this may affect outcomes; the duration of tinnitus should therefore be considered within trial data analysis plans. In terms of the risk of bias in the included studies, a major issue was under-reporting, such that the risk of bias was unclear in most cases, despite the availability of trial registrations, published protocols, and published study reports. Future reporting should follow established reporting guidelines (e.g., CONSORT [72]) and be mindful of the various sources of bias that trials are judged on, such as randomisation techniques, allocation concealment, blinding, and reporting [73]. In terms of outcomes, triallists should also consider the importance of core outcome sets. These are increasingly required by research funders and publishers and represent outcomes that are the most important to patients and other key stakeholders in research. Core outcome sets for clinical trials of tinnitus interventions have recently been established and so should be included in the planned outcomes for any trial involving a sound-, psychology-, drug-, or electrical stimulation-based intervention [74,75]. In the absence of specific single tools that measures each core outcome, the TFI (used in just three studies reviewed here) is a good option, as it includes subscales that measure most core outcomes for tinnitus intervention trials. Whilst other measures of tinnitus symptom severity might be more appropriate to the nature of the intervention or hypothesised mechanism of action, we recommend including the TFI or another set of measures that capture core outcomes in every trial. We also recommend that triallists state the hypothesised mechanism of their tinnitus intervention and include a relevant objective or behavioural measure of that mechanism. Many interventions, including neuromodulation interventions, are developed and trialled without a clear hypothesised mode of action—some of which may be missing from this review. Explicitly targeting specific neurophysiological mechanisms of tinnitus, and demonstrating a sustained change in neurophysiological activity, will be key to developing novel effective treatments in this exciting field.

## Figures and Tables

**Table 1:** Summary of included studies in review of neuromodulation treatments for tinnitus grouped by colour according to form of treatment.

	Participants	Intervention	Comparison	Main Outcome
Acoustic				
Tass 2012 [45]	44 male, 19 female. Chronic (≥6 months) tonal tinnitus. Mean age 45.7 years (SD = 10.8) in intervention and 57.6 years (SD = 6.3) in control group.	Acoustic CR(r) neuromodulation (4 tones per sequence above and below dominant tinnitus pitch) for 4–6 h a day for 12 weeks.	Placebo sound stimulation using tones distant from the dominant tinnitus pitch. Maximum of 1 h per day, for 12 weeks.	Tinnitus symptom severity
Hall 2022 [35]	46 male and 54 female. Chronic subjective tinnitus for >3 months. Mean age was 49.1 years (SD 11.3) in intervention group and 51.8 (SD 12.2) in control group.	Acoustic neuromodulation, 6 h per day for 12 weeks (blinded), then 4 h per day for 24 weeks (unblinded).	Placebo sound 6 h per day for 12 weeks (blinded), then 4 h per day for 24 weeks (unblinded).	Tinnitus symptom severity.
Theodoroff 2021 [47]	38 male and 23 female. Tonal tinnitus for >6 months. Mean age 53.9 (SD = 12.6) in intervention group and 55.8 (SD = 10.4) in control group.	Desyncra™ (Bad Neuenahr, Germany) 4–6 h per day for ~24 weeks.	Cognitive behavioural therapy, 6 sessions × 60 min over 8 weeks.	Tinnitus symptom severity.
Stein 2016 [44]	67 male and 33 female. Chronic (≥3 months), tonal tinnitus. Mean age 47.7 (SD 9.9) in the intervention and 47.1 (SD 11.7) in control group.	Tailor-made notched music (set according to tinnitus pitch). Participants listened to music 2 h per day for 12 weeks.	Placebo notched music (moving notch). Participants listened to music 2 h per day for 12 weeks.	Tinnitus symptom severity.
Smeele 2022 [43]	17 male and 6 female. Chronic (≥3 months), tonal tinnitus. Mean age 57.4 (SD = 16.4) in the intervention and 59.9 (SD = 8.5) in control group.	High-definition infraslow pink noise stimulation, 12 × 30 min over 4 weeks.	Acti-sham, stimulation shunted to induce negligible electric field in target areas, 12 × 30 min over 4 weeks.	Feasibility, safety, resting-state EEG.
transcranial Direct Current Stimulation				
Abtahi 2018 [28]	46 male, 23 female. Tinnitus > 1 year Mean age 47.4 years and 46.9 (SD = 14.6) in intervention groups and 45.4 (SD = 13.2) in control group.	tDCS (anodal or cathodal) one session of 2 mA stimulation for 20 min.	Sham stimulation. One session of 2 mA stimulation for 20 min. Dose not described.	Tinnitus intensity on a VAS.
Cardon 2022 [29]	43 males, 34 females. Chronic (>6 months) subjective tinnitus. Mean age 53.47 (14.39) in intervention and 51.95 (13.87) in control group.	High-definition tDCS, 6 × 30 min sessions over 3 weeks, each at 2 mA for 30 min.	Sham stimulation, 6 × 30 min sessions over 3 weeks, but constant current was only applied for the first 20 s.	Tinnitus symptom severity.
Cavalcanti 2015 [30]	9 males, 9 females with sensorineural hearing loss and chronic tinnitus. Mean age of all 54.72 years.	tDCS, 2 mA stimulation for 20 min daily for 5 days.	Sham stimulation, 20 min daily for 5 days but with only 10 s initial stimulation.	Tinnitus symptom severity.
da Silva Souza 2020 [31]	8 males, 16 females. Chronic tinnitus ≥ 6 months. Mean age 44.58 years (SD = 16.20) in intervention and 50.50 (SD = 9.72) in control group.	tDCS, 2 mA current for 20 min daily for 5 days.	Sham stimulation, 20 min daily for 5 days but with only 30 s initial stimulation.	Tinnitus symptom severity.
Dobie 1986 [32]	15 males, 5 females. Sensorineural hearing loss and tinnitus severe enough to justify participation. Mean age of all 50.25 years (SD = 12.26).	tDCS, 60 kHz carrier frequency modulated by a continuously swept 3 V signal across 5 kΩ load, ≤5 h per day for 7 days.	Sham stimulation. Device not connected to current. ≤5 h per day for 7 days.	Self-reported use and improvements (% scales).
Forogh 2016 [33]	14 males, 8 females. Chronic tinnitus (≥6 months). Mean age 49.8 years (SD = 4.1) in intervention and 46.6 years (SD = 5.3) in control group.	tDCS, 2 mA stimulation for 20 min daily for 5 days.	Sham stimulation, 20 min daily for 5 days but with only 30 s initial stimulation.	Tinnitus symptom severity.
Garin 2018 [34]	15 males, 5 females. Stable tinnitus for at least 2 months. Mean age of all was 50.9 years (SD = 12.9)	tDCS. Anodal or cathodal, 1 mA stimulation for 20 min.	Sham stimulation, 110 uA over 15 ms delivered every 550 ms over 20 min.	Self-report of improvement (single-item scales).
Mares 2022 [38]	23 males, 16 females. Mean age 49 (SD = 16.73) in intervention and 46.15 years (SD = 18.5) in control group.	tDCS, 1.5 mA for 20 min, 6 times over 2 weeks.	Sham stimulation. Device-specific preprogrammed sham protocol.	Tinnitus symptom severity.
Pal 2015 [40]	24 males, 18 females. Chronic (≥1) year non-pulsatile subjective tinnitus. Mean age 51.6 years (SD = 12.2) in intervention and 48.0 (SD = 9.9) in control group.	tDCS, 2 mA stimulation for 20 min daily for 5 days.	Sham stimulation, 20 min daily for 5 days but with only 90 s initial stimulation at 1 mA.	Tinnitus symptom severity.
Shekhawat 2014 [41]	36 males, 4 females. Chronic tinnitus (>2 years). Mean age 59.9 years (SD = 9.6) in intervention and 58.5 (SD = 6.4) in control group.	tDCS, 2 mA current, 20 min per day over 5 days.	Sham stimulation, 20 min daily for 5 days but with only 30 s initial stimulation at 2 mA.	Tinnitus symptom severity.
Shekhawat 2018 [42]	13 males. Chronic tinnitus (>2 years). Mean age of all 53.6 years (SD 11.7).	High-definition tDCS, 2 mA current for a single 20-min session.	Sham stimulation, 20-min session but with only 30 s initial stimulation at 2 mA.	Tinnitus symptom severity.
To 2017 [48]	22 males, 18 females. Chronic tinnitus (>1 year). Mean age 49.17 years (SD = 13.32) and 47.29 years (SD = 9.15) in interventions groups and 48.64 years (SD = 10.49) in control group.	tDCS, 1.5 mA for 20 min per session, two sessions per week for 4 weeks, with or without the addition of transcranial random noise stimulation.	Waiting list.	Tinnitus symptom severity.
Yadollahpour 2017 [50]	19 males, 23 females. Chronic tinnitus (≥6 months). Mean age 44.68 years (SD = 6.87) in the intervention and 47.53 years (SD = 7.56) in the control group.	tDCS, 4 mA current (maximum output) for 20 min per day over 5 days.	Sham stimulation, 20 min session per day for 5 days but with only 30 s initial stimulation at (max) 4 mA.	Tinnitus symptom severity.
Yadollahpour 2019 [51]	Sex not reported. Chronic intractable tinnitus. Mean age of all 46.5 years (SD = 1.4).	tDCS (single session) anodal or cathodal. 2 mA current for 20 min.	Sham stimulation. 20-min session but with only 30 s initial stimulation at 2 mA.	Tinnitus symptom severity.
Teismann 2014 [46]	Sex not reported. Chronic (≥3 months), tonal tinnitus. Mean age 42.9 years (SD = 6.87) and 44.45 years (SD = 13.29) in intervention groups and 44.91 years (SD = 9.92) in control group.	tDCS Anodal or cathodal, 2 mA for 30-min sessions over 5, paired with notched music which also was listed to for 2 h post tDCS.	Sham stimulation. 30-min session but with only 30 s initial stimulation at 2 mA.	Tinnitus symptom severity.
Vagus nerve				
Tyler 2017 [49]	25 males, 5 females. Tinnitus for ≥1 year. Mean age 55.9 years (SD = 7.6) in intervention and 54.9 years (SD = 9.1) in control group.	Vagus nerve stimulation, 15 × 0.8 mA pulses every 30 s for 2.5 h paired with tones. Daily for 6 weeks.	Vagus nerve stimulation not paired with tones, 2.5 h daily for 6 weeks.	Tinnitus symptom severity.
Mei 2014 [39]	29 males, 34 females. Tinnitus recurrent over 1 month or persistent for ≥5 days. Mean age of all 41.1 years (SD = 12.6).	Auricular acupuncture and sound masking, 1 mA current, pulse frequency of 20 Hz, pulse width of 1 ms; 20 min, twice per day for 8 weeks.	Flunarizine hydrochloride (5 mg orally before bed), and oryzanol 20 mg orally three times per day, for 8 weeks.	Tinnitus symptom severity.
transcranial Alternating Current Stimulation				
Lee 2014 [37]	39 males, 26 females. Subjective, unilateral tinnitus > 6 months. Mean age 46.6 years (SD = 13.0) in intervention and 45.6 (SD = 11.0) in control group.	Alternating current stimulation, 15 mA stimulation for 30 s at 5 sites on external earl 8 sessions over 4 weeks.	Sham stimulation. Identical sessions but power supply switched off.	Tinnitus symptom severity.
Bimodal				
Jones 2023 [36]	59 males, 49 females. Tinnitus > 6 months. Mean age 47.0 (SD = 1.8) in intervention and 47.1 (SD = 1.8) in control group.	Bisensory (auditory and somatosensory) stimulation, 3 biphasic squarewave pulses; 150 microseconds per phase paired with auditory stimulation, 30 min daily for 6 weeks	Auditory stimulation only, 30 min daily for 6 weeks.	Tinnitus symptom severity.

EEG = encephalography. ms = milliseconds. tDCS = transcranial direct current stimulation. VAS = Visual Analogue Scale.

## Data Availability

This review is a synthesis of previously published data.

## Appendix A

Box A1 Example full search strategy. Search: **tinnitus AND (tdcs OR neuromodulation OR coordinated reset OR desynchronization OR ((transcranial OR transcutaneous) AND stimulation) OR electrostimulation OR neurostimulation)** (“tinnitus” [MeSH Terms] OR “tinnitus” [All Fields]) AND (“transcranial direct current stimulation” [MeSH Terms] OR (“transcranial” [All Fields] AND “direct” [All Fields] AND “current” [All Fields] AND “stimulation” [All Fields]) OR “transcranial direct current stimulation” [All Fields] OR “tdcs” [All Fields] OR (“neuromodulate” [All Fields] OR “neuromodulating” [All Fields] OR “neuromodulation” [All Fields] OR “neuromodulations” [All Fields] OR “neuromodulative” [All Fields] OR “neurotransmitter agents” [Pharmacological Action] OR “neurotransmitter agents” [MeSH Terms] OR (“neurotransmitter” [All Fields] AND “agents” [All Fields]) OR “neurotransmitter agents” [All Fields] OR “neuromodulator” [All Fields] OR “neuromodulators” [All Fields]) OR ((“coordinate” [All Fields] OR “coordinated” [All Fields] OR “coordinately” [All Fields] OR “coordinates” [All Fields] OR “coordinating” [All Fields] OR “coordination” [All Fields] OR “coordinations” [All Fields] OR “coordinative” [All Fields] OR “coordinatively” [All Fields] OR “coordinator” [All Fields] OR “coordinator s” [All Fields] OR “coordinators” [All Fields]) AND (“reset” [All Fields] OR “resets” [All Fields] OR “resetting” [All Fields] OR “resettings” [All Fields])) OR (“desynchronisation” [All Fields] OR “desynchronised” [All Fields] OR “desynchronization” [All Fields] OR “desynchronizations” [All Fields] OR “desynchronize” [All Fields] OR “desynchronized” [All Fields] OR “desynchronizes” [All Fields] OR “desynchronizing” [All Fields] OR “desynchronous” [All Fields]) OR ((“transcranial” [All Fields] OR “transcranially” [All Fields] OR (“transcutaneous” [All Fields] OR “transcutaneously” [All Fields])) AND (“stimulate” [All Fields] OR “stimulated” [All Fields] OR “stimulates” [All Fields] OR “stimulating” [All Fields] OR “stimulation” [All Fields] OR “stimulations” [All Fields] OR “stimulative” [All Fields] OR “stimulator” [All Fields] OR “stimulator s” [All Fields] OR “stimulators” [All Fields])) OR (“electrostimulated” [All Fields] OR “electrostimulating” [All Fields] OR “electrostimulation” [All Fields] OR “electrostimulations” [All Fields] OR “electrostimulator” [All Fields] OR “electrostimulators” [All Fields]) OR (“neurostimulation” [All Fields] OR “neurostimulations” [All Fields] OR “neurostimulator” [All Fields] OR “neurostimulators” [All Fields]))
**Translations**
**tinnitus:** “tinnitus” [MeSH Terms] OR “tinnitus” [All Fields] **tdcs:** “transcranial direct current stimulation” [MeSH Terms] OR (“transcranial” [All Fields] AND “direct” [All Fields] AND “current” [All Fields] AND “stimulation” [All Fields]) OR “transcranial direct current stimulation” [All Fields] OR “tdcs” [All Fields] **neuromodulation:** “neuromodulate” [All Fields] OR “neuromodulating” [All Fields] OR “neuromodulation” [All Fields] OR “neuromodulations” [All Fields] OR “neuromodulative” [All Fields] OR “neurotransmitter agents” [Pharmacological Action] OR “neurotransmitter agents” [MeSH Terms] OR (“neurotransmitter” [All Fields] AND “agents” [All Fields]) OR “neurotransmitter agents” [All Fields] OR “neuromodulator” [All Fields] OR “neuromodulators” [All Fields] **coordinated:** “coordinate” [All Fields] OR “coordinated” [All Fields] OR “coordinately” [All Fields] OR “coordinates” [All Fields] OR “coordinating” [All Fields] OR “coordination” [All Fields] OR “coordinations” [All Fields] OR “coordinative” [All Fields] OR “coordinatively” [All Fields] OR “coordinator” [All Fields] OR “coordinator’s” [All Fields] OR “coordinators” [All Fields] **reset:** “reset” [All Fields] OR “resets” [All Fields] OR “resetting” [All Fields] OR “resettings” [All Fields] **desynchronization:** “desynchronisation” [All Fields] OR “desynchronised” [All Fields] OR “desynchronization” [All Fields] OR “desynchronizations” [All Fields] OR “desynchronize” [All Fields] OR “desynchronized” [All Fields] OR “desynchronizes” [All Fields] OR “desynchronizing” [All Fields] OR “desynchronous” [All Fields] **transcranial:** “transcranial” [All Fields] OR “transcranially” [All Fields] **transcutaneous:** “transcutaneous” [All Fields] OR “transcutaneously” [All Fields] **stimulation:** “stimulate” [All Fields] OR “stimulated” [All Fields] OR “stimulates” [All Fields] OR “stimulating” [All Fields] OR “stimulation” [All Fields] OR “stimulations” [All Fields] OR “stimulative” [All Fields] OR “stimulator” [All Fields] OR “stimulator’s” [All Fields] OR “stimulators” [All Fields] **electrostimulation:** “electrostimulated” [All Fields] OR “electrostimulating” [All Fields] OR “electrostimulation” [All Fields] OR “electrostimulations” [All Fields] OR “electrostimulator” [All Fields] OR “electrostimulators” [All Fields] **neurostimulation:** “neurostimulation” [All Fields] OR “neurostimulations” [All Fields] OR “neurostimulator” [All Fields] OR “neurostimulators” [All Fields]
